# The Role of Diet and Gut Microbiota in Regulating Gastrointestinal and Inflammatory Disease

**DOI:** 10.3389/fimmu.2022.866059

**Published:** 2022-04-05

**Authors:** Paul A. Gill, Saskia Inniss, Tomoko Kumagai, Farooq Z. Rahman, Andrew M. Smith

**Affiliations:** ^1^ Department of Microbial Diseases, UCL Eastman Dental Institute, University College London, London, United Kingdom; ^2^ Department of Gastroenterology, University College London Hospitals National Health Service (NHS) Foundation Trust, London, United Kingdom

**Keywords:** diet, inflammation, gut microbiota, gastrointestinal tract, inflammatory bowel disease, mucosal immunity, fermented (cultured) dairy products

## Abstract

Diet is an important lifestyle factor that is known to contribute in the development of human disease. It is well established that poor diet plays an active role in exacerbating metabolic diseases, such as obesity, diabetes and hypertension. Our understanding of how the immune system drives chronic inflammation and disease pathogenesis has evolved in recent years. However, the contribution of dietary factors to inflammatory conditions such as inflammatory bowel disease, multiple sclerosis and arthritis remain poorly defined. A western diet has been associated as pro-inflammatory, in contrast to traditional dietary patterns that are associated as being anti-inflammatory. This may be due to direct effects of nutrients on immune cell function. Diet may also affect the composition and function of gut microbiota, which consequently affects immunity. In animal models of inflammatory disease, diet may modulate inflammation in the gastrointestinal tract and in other peripheral sites. Despite limitations of animal models, there is now emerging evidence to show that anti-inflammatory effects of diet may translate to human gastrointestinal and inflammatory diseases. However, appropriately designed, larger clinical studies must be conducted to confirm the therapeutic benefit of dietary therapy.

## 1 Introduction

Diet is an important lifestyle factor that can contribute to the development of human disease, particularly as poor diet contributes to the development of metabolic diseases, such as obesity, diabetes, and hypertension (1). These may be driven by underlying inflammation, a tightly regulated immune process, whereby both specialised immune and non-immune cells release inflammatory mediators, cytokines, and chemokines in response to a pathogen or tissue damage. This inflammatory cascade drives subsequent recruitment of leucocytes to the site of infection, to contain and eliminate the infection, clear tissue damage, and eventually initiate resolution of inflammation. However, if the initial inflammatory stimulus cannot be cleared, a state of chronic inflammation may develop with ensuing pathology. A wide array of metabolic and immune diseases have now been linked to a defective inflammatory response (2–4).

Interactions between the immune system, inflammation, and diet in driving human metabolic disease have been described (5). However, the contribution of dietary factors to inflammatory conditions are poorly defined. Our understanding of the interactions between dietary factors and gut microbiota that regulate immune mechanisms is also in its relative infancy. It is also important to consider gut and broader host physiology that dictate how nutrients interact with the body and the microbes that reside within the gastrointestinal tract. This review aims to outline the evidence that diet may regulate inflammation that drive human gastrointestinal and inflammatory disease, both directly by modulating the immune system and indirectly by interacting with gut microbiota. In addition, we will assess the current and emerging body of evidence for using dietary therapy to treat these conditions, outlining current challenges that must be addressed to achieve translation into clinical use.

## 2 Epidemiological Associations Between Dietary Patterns and Inflammatory Disease

A western diet, characterised by high intake of fats and carbohydrates derived from refined sugars and processed food with reduced consumption of dietary fibre and whole grains, has been linked to inflammatory disease (6). This observation initially derived from epidemiological studies, that compared overall dietary patterns and disease incidence across the world. For example, incidence rates of asthma and inflammatory bowel disease are higher in westernised countries when compared to non-westernised countries that consume alternative diets (7, 8). However, these comparisons may not account for other confounding environmental and lifestyle factors. As such, studies of dietary patterns in large groups living from similar regions provide better evidence of a connection between diet and inflammation. Assessment of dietary patterns using diet quality scores estimated from food frequency questionnaires highlight that those who have higher dietary scores and report consuming healthier foods (e.g. whole grains, nuts/legumes, fruits, and vegetables), are less likely to develop inflammatory conditions or experience symptoms of disease (9, 10). In contrast, those who frequently consume processed foods high in refined sugars, fats, and oils, record low diet quality scores that are associated with higher prevalence of disease and display exacerbated clinical disease scores (11, 12). Low dietary quality scores are also associated with higher levels of plasma IL-6, E-selectin and soluble ICAM-1 in healthy people suggesting that a poor diet may promote a state of subclinical chronic inflammation (13). A poor diet quality may also predispose individuals to infection, with poor diet quality scores related to a higher incidence and severity of COVID-19 (14).

To specifically investigate the relationship between dietary intake, inflammatory markers and the development of disease, dietary inflammation scores have also been used to estimate the overall inflammatory potential of a diet. Measurements such as the dietary inflammatory index and empirical dietary inflammatory pattern (EDIP) incorporate data from association studies in healthy cohorts linking consumption of food components with levels of inflammatory cytokine such as IL-6, IL-1β, IL10, TNF-α and C-reactive protein (CRP) (15, 16). Consumption of foods high in cholesterol, sugars, and saturated fats (e.g. processed meats, red meats, soft drinks) are associated with higher levels of CRP and IL-6 (15–17). In contrast, consumption of foods containing fibre, vitamins, low levels of alcohol, herbs, and spices (e.g., leafy green and root vegetables, fruits, wine) are associated with lower CRP and IL-6 (15, 16, 18). However, the magnitudes of these associations are small and may be confounded by adiposity levels.

Compared to healthy individuals, subjects with asthma have been found to consume a diet with a higher dietary inflammatory score, which was associated with a more severe phenotype. Furthermore, reduced forced expiratory volume (FEV_1_) was directly correlated with an increased dietary inflammatory index score, as well as increased serum IL-6 (19). A large study involving over 200,000 individuals and using EDIP scores interestingly revealed an increased risk of developing Crohn’s disease, but not ulcerative colitis, the other major form of inflammatory bowel disease (IBD) (20). Those with EDIP scores in the highest quartile were found to have a 51% increased risk. This association remained after adjusting for dietary fibre suggesting that other nutrients were involved. An association with ultra-processed foods (i.e., packaged foods containing food additives, artificial flavours, and colours) has also been observed, with higher daily intake associated with increased risk of IBD, particularly for those who consume >5 serves/day (21). Intake of soft-drinks and processed meats were also identified as increasing risk in this cohort. Despite significant correlation with human inflammatory disease, there are limitations in drawing conclusions from dietary inflammation scores: they only consider cytokine levels to define inflammation; include data from *in vitro* studies and consumption data is restricted to individual food groups to calculate the overall inflammatory score.

A range of alternative dietary patterns have been proposed as having anti-inflammatory properties. These generally differ from that of an established western diet in that they are low-fat and high fibre, with limited consumption of processed foods. Features of these diets and associations with immune parameters within healthy populations are summarized in **Table 1**. Consumption of these dietary patterns is generally linked to reduced blood inflammatory markers in healthy people and may also be protective against development of inflammatory disease as suggested by higher rates of allergy, asthma and IBD in migrants who adopt a western diet when moving from a country with a traditional diet (40, 41). Increased mucosal intraepithelial lymphocytes and lamina propria macrophages were observed in colonic biopsies taken from native Africans after 29 days of consuming a western-style American diet (30). This study supports the notion of a rapidly induced alteration in mucosal immunity following a major change in diet, with a particularly strong impact on the colon.

**Table 1:** Associations between dietary patterns and immune parameters from healthy cohorts.

Dietary pattern	Countries associated	Foods	Nutrient characteristics	Immune parameters	Ref.
Western diet	UK, USA, Canada, Australia, Mexico	Processed foods, refined sugars, refined grains Low fruit & vegetable consumption	High saturated fat, carbohydrate, salt, cholesterol Low fibre, vitamins, minerals	↑ serum CRP, IL-6, E-selectin, sICAM-1, sVCAM-1 ↑ platelet CD41, platelet-granulocyte aggregates ↑ colonic *IL1B, FAS, TNF, IFNAR1, STAT2* expression	(6, 22)(23, 24)
Mediterranean diet	Italy, Greece, Cyprus,	Fish, cheese, yoghurt, cereals, fruits & vegetables, wine, olive oil Meat, milk	Low saturated fat, high monounsaturated fat intake High fibre, high vitamin B, C, E and polyphenols, moderate ethanol intake	↓ serum IL-6, CRP, TNF-α, ICAM-1	(25–27)
Indigenous African diet	Burkina Faso, Tanzania, South Africa,	unrefined grains, legumes, vegetables	High fibre, resistant starch, plant-derived proteins low animal protein intake	↓ plasma IL-1β ↓ IFN-γ, TNF, IL-6 in response to whole blood LPS stimulation ↓ macrophages in lamina propria	(28, 29)(30)
Traditional East-Asian diet	Japan, Korea	Fermented vegetables, soy, rice, fish	High salt, carbohydrate, sodium low fat	↑ plasma IL-10 ↓ serum IL-6, CRP	(31, 32)(33, 34)(35)
Plant-based diet	n/a	Whole grains, cereals, fruits, vegetables, legumes, nuts, low red meat consumption	High fibre, plant-derived proteins, fats polyphenols low/no animal derived protein or fats	↓ serum CRP, ↓ overall WBC, ↓ blood neutrophils, monocytes	(36, 37)(38, 39)

LPS, Lipopolysaccharide; WBC, White blood cell count.

↑, increased; ↓, decreased; n/a, not applicable.

However, aspects of traditional dietary patterns may also promote inflammation if consumed in excess. Alcohol consumption above a 30 g/day moderate threshold was found to significantly correlate with increased serum inflammatory markers in those who followed a Mediterranean diet (25, 42). The high levels of sodium intake (>3200 mg/day) in traditional Japanese diets may also promote inflammation and exacerbate kidney disease, as occurs in mice fed a high-salt diet in models of kidney disease (43–45). Plant-based diets may also require additional supplementation to ensure adequate intake of micronutrients such as vitamin B12, calcium, zinc and niacin that are crucial for immune cell function (46, 47). Furthermore, anti-inflammatory properties of alternative dietary patterns likely involve additional non-nutritional factors, such as meal-timing. The circadian clock exerts an influence on the immune system, as those with lifestyle patterns that cause chronic disruption of the circadian rhythm (e.g. shift workers) have increased susceptibility to inflammatory and metabolic diseases (48). Circadian misalignment by mistimed feeding and sleeping resulted in upregulated pro-inflammatory cytokine signalling and down-regulated antigen presentation in healthy individuals (49). Furthermore, higher levels of salivary IL-6 and CRP were observed in Spanish children who consumed their evening meal later in the evening (after 21:00), when compared to those who ate earlier in the evening (50).

## 3 Nutrient-Immune System Interactions in Inflammatory and Gastrointestinal Diseases

The optimal functioning of the immune system is highly dependent on a balanced and adequate diet. Specifically, both deficiency or excess of certain nutrients can adversely affect immune system function, and in turn are linked to inflammatory diseases. This section will review the direct effects of both micronutrients and macronutrients on the immune system, and their potential link to risk of inflammatory diseases.

### 3.1 Dietary Fats

**Figure 1:**
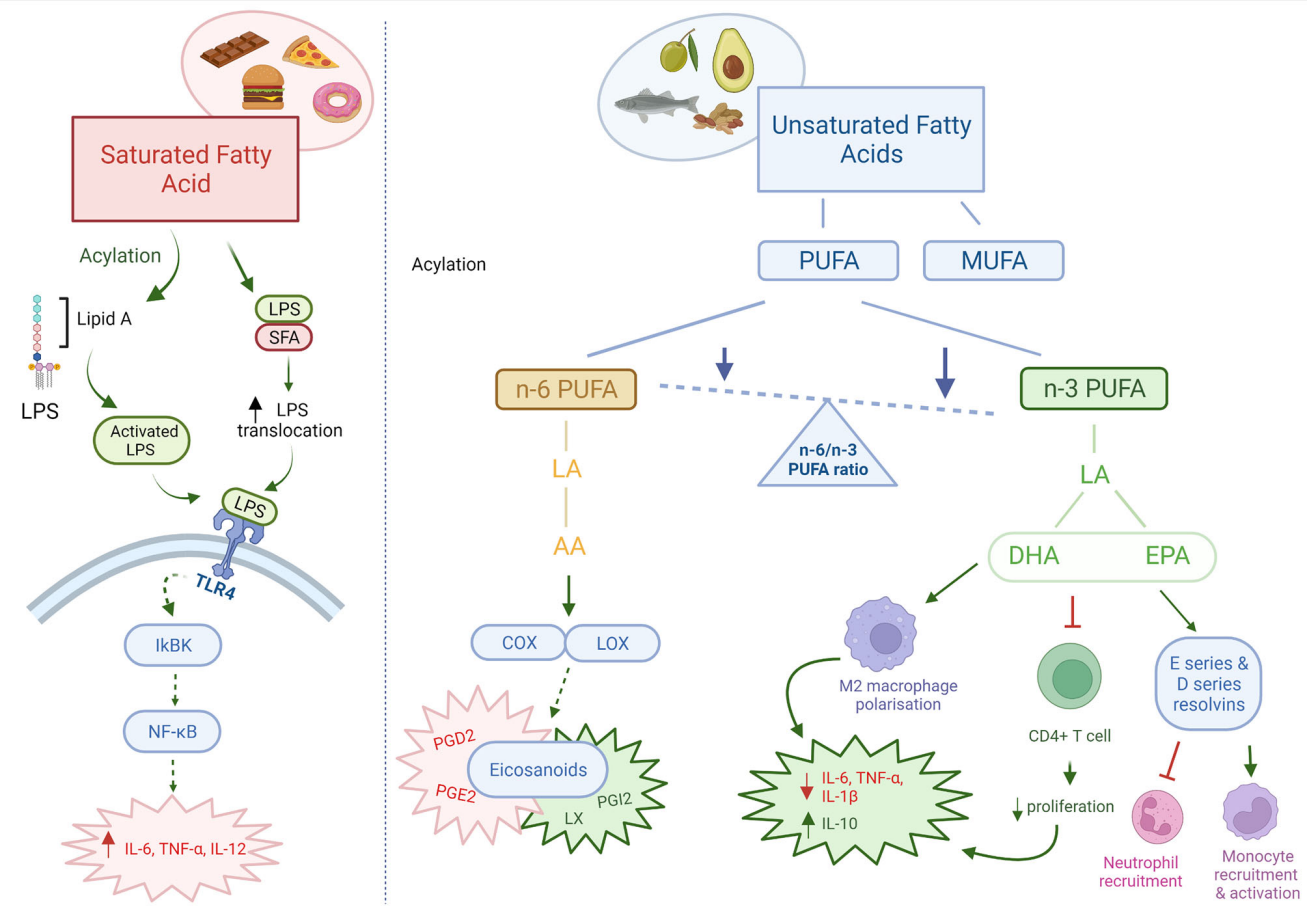
Pro and anti-inflammatory effects of dietary fats. Dietary fats directly and indirectly act as both pro-inflammatory and anti-inflammatory mediators. Saturated fatty acids are pro-inflammatory in nature, through increased translocation and activation of LPS leading increased TLR4 signalling. n-3 PUFAs may be immunosuppressant through its effects on immune cells and intestinal barrier integrity. n-6 PUFAs are mainly pro-inflammatory, however can also produce anti-inflammatory eicosanoids. The ratio of n-6/n-3 PUFAs are important in determining the inflammatory state in the body. Red: pro-inflammatory; Green: anti-inflammatory; Orange: pro- & anti-inflammatory. AA, arachidonic acid; PUFA, polyunsaturated fatty acid; MUFA, monounsaturated fatty acids; LA, linoleic acid; DHA, docosahexaenoic acid; EPA, eicosapentaenoic acid; COX, cyclooxygenase; LOX, lipoxygenase; PG, prostaglandin; LX, lipoxin. Created with BioRender.com.

Dietary fats are an essential energy source for the body, and a fundamental part of the structure of immune cells, therefore playing a key role in modulating the immune response in health and disease (51). Dietary fats may also contribute to the levels and composition of adipose tissue. Indeed, increased adipose tissue contributes to low grade inflammation characterised by enhanced secretion of pro-inflammatory cytokines, as extensively reviewed elsewhere (52). A reduction in total fat intake in men from 30% to 25% was shown to increase T and B cell proliferation and circulatory numbers (53). In addition, decreasing total fat intake by 10% was associated with an increase in the activity of natural killer (NK) cells (54, 55). Besides the effects of total fat intake, current research suggests that the type of fat consumed is of particular importance (**Figure 1**). Saturated and unsaturated fatty acids regulate immune function by acting through surface G-protein coupled receptors, intranuclear receptors and altering membrane composition and fluidity (56).

An imbalance between saturated and unsaturated (omega-6 and omega-3) fatty acids, has a significant effect on the immune homeostasis, and have been associated with an increased risk of developing atherosclerosis, coronary heart disease, obesity and metabolic syndrome. It is generally accepted that saturated fatty acids promote an inflammatory response by activating Toll-like receptor 4 (TLR4) and promoting pro-inflammatory cytokine production (57). Conversely, omega-3 polyunsaturated fatty acids (n-3 PUFA) obtained from fish and plant-based dietary sources have anti-inflammatory effects (58). The mechanisms of action for n-3 PUFA immunosuppressive effects may be *via* their interaction with T cell signalling, effects on the intestinal barrier and/or direct effects on pro-inflammatory cytokine production (59–62). An *in vitro* model showed that stimulation of T84 intestinal epithelial cells with n-3 PUFAs eicosapentaenoic acid (EPA) and docosahexaenoic acid (DHA) restored the intestinal barrier integrity after impairment (63). Supplementation of n-3 PUFA in dextran sulfate sodium (DSS) induced colitis in mice have been shown to be effective in alleviating disease activity, by reducing the infiltration of inflammatory cells and the production of TNF-α and IL-6 (64). In addition, n-3 PUFA stimulated the polarization of anti-inflammatory M2 macrophages and suppressed the polarization of pro-inflammatory M1 macrophages in mice (65). These effects of n-3 PUFA on macrophages were associated with the inhibition of multiple kinases, including: IκB kinase, Akt, and focal-adhesion kinase. In both human and animal models, n-3 PUFA supplementation may also lead to remission of inflammatory bowel disease (66–69). Whilst the effects of monounsaturated fatty acids (MUFAs) are not as well documented, they have also been shown to have anti-inflammatory properties. In an obesity animal model, a diet rich in MUFAs for 8 weeks was found to increase the expression of anti-inflammatory mediators, such as IL-14 and IL-10, and increase the M2 macrophage levels (70). In human studies, diets rich in MUFAs have been associated with an improved inflammatory profile, reduced CRP levels, improved insulin sensitivity and decreased risk of developing cardiovascular disease (71–73).

In contrast to n-3 PUFA, the consumption of n-6 PUFA, mainly obtained from oils, meats and cereal based products, increases the number of eicosanoid inflammatory mediators, including prostaglandins and leukotrienes, which in turn can promote cytokine production and the activity of inflammatory cells (58). Whilst, most eicosanoids are pro-inflammatory, the lipoxygenase derivative lipoxin has been found to have anti-inflammatory properties important for resolution of inflammatory responses (74). A large European cohort study found a significant correlation between dietary linoleic acid (n-6 PUFA) intake and development of ulcerative colitis (75). In contrast, dietary intake of the shorter chain n-3 PUFA DHA was found to be protective against developing ulcerative colitis. Studies suggest that a reduced omega 6:omega 3 ratio is associated with an attenuated inflammatory response, and reduced release of IL-6 (76, 77). This may be impaired in those who carry single nucleotide polymorphisms (SNPs) in genes involved in PUFA metabolism. Children who consume a diet with a higher ratio of n-6:n-3 PUFA intake may be more susceptible to Crohn’s disease if they also carry SNPs in Cytochrome P450 Family 4 Subfamily F Member 3 (CYP4F3), Fatty Acid Desaturase 1 and 2 (FADS1, FADS2) (78). However, both a large European study and Cochrane review have reported that n-3 PUFA are ineffective in the maintenance and remission of Crohn’s disease (79, 80). Furthermore, a recent study demonstrated that PUFAs induce gut inflammation and potentially exacerbate Crohn’s disease (81). In patients with active Crohn’s disease, n-3 and n-6 PUFA instigated epithelial chemokine expression and a systemic inflammatory stress signature. The levels of PUFA ingested by the Crohn’s disease patients correlated with clinical and biochemical disease activity. Taken together, more research is needed to confirm the effects of both n-6 and n-3 PUFA on intestinal inflammation.

### 3.2 Dietary Protein

It is well established that protein deficiency impairs immune function and increases susceptibility to infectious and inflammatory diseases (82). Several human studies have reported a link between high dietary protein intake and increased risk of IBD and IBD relapse (83–85). Jantchou et al. reported that the consumption of a high protein diet was associated with an increased risk of IBD. In addition, the concentration of dietary protein may affect colitis development: mice consuming a high protein diet had increased colitis severity compared to mice consuming a low protein diet (86). Interestingly, other colitis mice models have shown that a high protein or moderate-high protein diet could be beneficial in post-colitis epithelial repair and mucosal healing, respectively (87, 88). The source of the protein may also be important, with high animal protein consumption from meat or fish, but not of eggs or dairy products associated with increased IBD risk. Murine colitis models have shown that a diet high in red meat worsens disease activity index compared to a casein-based protein diet (89). However, it is not possible to exclude the effects of other meat components such as heme. In contrast, plant-based soybean protein have been shown to have anti-inflammatory effects, particularly in combination with isoflavones (a polyphenol present in soy) (90–92). Soy protein in its isolated form is not as well studied, however one murine study showed that supplementation with soy protein isolate for 5 weeks resulted in the inhibition of NF-κB and blocked the secretion of pro-inflammatory cytokines (93). In addition, soy protein has been found to have antioxidant effects *in vitro*, and moderate DSS-induced inflammation and loss of gut function *in vivo (* 94).

The role of protein in coeliac disease is well established. Gliadin, a gluten peptide, can trigger innate and adaptive immune responses that ultimately lead to coeliac disease in genetically susceptible individuals. Enterocyte damage happens rapidly after gluten exposure, with an increase in IL-2 observed in plasma from patients with coeliac disease 4 hours after gluten intake (95, 96). Upregulation of IL-15 in both the epithelium and the lamina propria is a hallmark of coeliac disease and correlates with the degree of mucosal damage seen in these patients (97–99). IL-15 acts on innate immunity through dendritic cells that drive T cells toward a Th1 response, leading to epithelial damage (100–102). Furthermore, gliadin may be presented by HLA-DQ-2/8 to CD4^+^ T cells, which results in a proinflammatory response, ultimately leading to hyperplasia, villous blunting and intestinal epithelial cell death (103, 104). Amylase trypsin inhibitor is another gluten protein that is not fully digested in the body and may promote an intestinal and extra-intestinal immune response by activating TLR4 (105, 106).

Specific amino acids have also been identified to modulate the immune system. Amino acids play an important role in plasma, including the activation of T and B lymphocytes, NK cells and macrophages. In addition, they regulate cellular redox state, gene expression and lymphocyte proliferation, as well as antibody and cytokine production (82). For example, arginine has been shown to enhance cellular immune mechanisms (particularly T cell function) and have immunosuppressant effects (107). Arginine supplementation results in enhanced T lymphocyte response and increased CD4^+^ T helper (Th) cells in postoperative cancer patients (108). Alanine is a major substrate for hepatic synthesis of glucose, which is a significant energy source for leukocytes (109). Further, studies show that alanine supplementation in mice prevents apoptosis, enhances cell growth, and increases plasma cell antibody production (110, 111). The amino acid glutamine is also a major energy source substrate for cells of the immune system, that can be converted to metabolites such as glutamate, alanine, lactate, and pyruvate. These substrates are necessary for activation, proliferation and activity of lymphocytes, macrophages, neutrophils and NK cells (82). Interestingly, supplementation with dietary arginine and glutamine significantly reduces colonic IL-17 and TNF-α in a DSS-induced colitis mice model (112). This was also associated with changes to colonic NF-κB, PI3K-Akt and MLCK signalling pathways, suggesting these amino acids have anti-inflammatory effects in the colon.

In addition, endogenously synthesised tryptophan metabolites (kynurenines, serotonin and melatonin) as well as bacterially-derived tryptophan metabolites (as reviewed below) have significant effects on the gut microbiota and host immune system (113–117). Dietary sources of tryptophan include milk, dried prunes, tuna, chicken and peanuts. Serotonin exhibits anti-inflammatory properties in rodents, regulating gut permeability and mucosal inflammation (118–120). Indeed, a recent study showed that elevated serotonin levels in mice inhibited autophagy and increased susceptibility to colitis, and elevated serotonin levels in humans were associated with worsening inflammation and Crohn’s disease flareups (121). In a large IBD cohort study, patients with active disease have increased levels of tryptophan metabolites, especially quinolinic acid, suggesting an increase in tryptophan degradation compared to controls (122). Further research is needed to elucidate if tryptophan deficiency could contribute to the development of IBD or aggravate disease activity.

### 3.3 Dietary Vitamins

Vitamins play an important role in regulating the immune system, as vitamin deficiencies can adversely affect the immune system and potentially lead to the development or aggravation of infectious and inflammatory diseases. For example, vitamin deficiencies are commonly found in IBD patients, particularly Crohn’s disease. However, little is known about whether these vitamin deficiencies are a consequence or risk factor for IBD (123). Many studies have highlighted the importance of vitamin D on the immune system and inflammatory response, especially the active form 1,25 dihydroxy vitamin D3 (124). Vitamin D deficiency is prevalent amongst patients with inflammatory diseases, such as: asthma, IBD, atherosclerosis and arthritis (125).

The beneficial effects of vitamin D on disease activity are thought to be mediated by the effects of vitamin D on the immune system. Vitamin D can modulate inflammatory responses on many different levels. This includes: the regulation of genes that generate pro-inflammatory mediators (e.g. COXs); its effects on transcription factors, such as NF-κB, which regulate inflammatory gene expression; and, the activation of signalling cascades which regulate the inflammatory response (124). The active form of vitamin D is generated *via* ligation of nuclear vitamin D receptor (VDR), that is present in immune cells and regulates cellular activity (126). For example, vitamin D has been shown to induce the production of antimicrobial peptides from neutrophils and macrophages (127–129). Other studies show that vitamin D can also enhance the antimicrobial activity of macrophages by increasing TLR and CD14 expression (130). Furthermore, it can regulate FOXP3^+^ regulatory T (Treg) cells by inducing their differentiation, in turn promoting the secretion of anti-inflammatory cytokines (131, 132).

Vitamin A also has a crucial role in optimal immune system function and is known for its anti-inflammatory properties (124). Whilst vitamin A is important in maintaining the integrity of gastrointestinal epithelium, it is also key in regulating the number and function of NK cells, macrophages, and neutrophils (133–136). Vitamin A regulates the differentiation of dendritic cell precursors and promotes secretion of pro-inflammatory IL-12 and IL-23 from these cells (135). In addition, vitamin A is involved in the antimicrobial action of macrophages, therefore playing an important role in the defence against pathogens (137). With regards to adaptive immunity, the vitamin A metabolite retinoic acid inhibits pro-inflammatory Th17 cells whilst promoting differentiation and maintenance of anti-inflammatory Treg cells in mice (138, 139). Vitamin A deficiency negatively affects B cell function and impairs antibody responses in mice (140, 141). Finally, vitamin A can activate T cells and influence the expression of membrane receptors that mediate T cell signalling in mice (142, 143). The supplementation of vitamin A has been shown to be beneficial in many diseases, including: broncho-pulmonary dysplasia and some forms of cancer (124). In contrast, whilst most studies support the benefits of vitamin A in IBD patients, some studies suggest that vitamin A stimulates the release of pro-inflammatory cytokines and aggravate disease activity in IBD (144). More extensive research is needed to confirm the mechanisms by which vitamin A influences systematic and intestinal inflammation.

### 3.4 Dietary Minerals

Many different minerals have been studied in relation to inflammation and the immune system. Both human and animal studies have shown that high salt diets can affect the immune response by increasing inflammatory macrophages and T cell responses, and supressing neutrophil-mediated immune responses (145). A high-salt environment induces Th17 cell responses by driving p38 MAPK pathway, which also exacerbates inflammation in a mouse model of multiple sclerosis (146). Mice fed high salt diets have enhanced expression of pro-inflammatory genes (e.g. Rac1, Map2K1) and suppressed cytokine and chemokine genes (Ccl3, Ccl4) in the colon and small intestine, resulting in more severe DSS and dinitrobenzene sulfonic acid induced colitis compared to mice fed control diets (147).

Additionally, the effects of iron homeostasis on immune function are well established (148, 149). Maintaining iron homeostasis is extremely important due to both the anti- and pro-inflammatory potential of iron. Iron is an essential mineral, that can be found in liver, red meat, beans and nuts, and has been shown to have multiple direct effects on the immune system by regulating cytokine production, generating reactive oxygen species (ROS) which kill pathogens and contributing to the differentiation and proliferation of T lymphocytes (150–152).

The immune system is also influenced by zinc, which can be found in animal products such as meat, fish, and eggs, and in smaller doses in whole grains and legumes. Zinc is an essential trace element required for critical cellular functions such as signal transduction, transcription, and differentiation. Consequently, zinc deficiency may cause dysregulation of inflammatory responses in immune cells, resulting in oxidative stress and increased release of pro-inflammatory cytokines (153, 154). In contrast, zinc supplementation inhibits the activation of NF-κB, resulting in decreased pro-inflammatory cytokine production. Furthermore, zinc may also inhibit allergen-induced proliferation of T cells and promote differentiation toward Treg cells (155). Indeed, human studies have shown that consumption of 45 mg/day of zinc supplements has been shown to be effective in decreasing cytokine responses in mononuclear cells stimulated ex vivo (156) Consumption of zinc supplements may also inhibit viral activity, which may shorten the duration of a cold (157, 158).

Selenium, a trace element mainly found in bread, cereals, meat, fish and dairy products, is also closely linked to immunity. Studies have shown that selenium deficiency is common in IBD, rheumatoid arthritis and coeliac disease (159–161). In coeliac disease, selenium has been recommended as a therapeutic measure to block IL-15, in turn decreasing epithelial damage and preventing extra-intestinal complications (162). Human studies show that low selenium levels are associated with increased CRP levels and increased products of reactive oxygen species that cause tissue damage and organ failure (163, 164). Selenium is also a cofactor in 25 different selenoproteins, of which the antioxidant glutathione peroxidase has critical functions in maintenance of intestinal mucosal homeostasis (165, 166).

### 3.5 Dietary Polyphenols

Polyphenols are naturally occurring compounds in fruits, vegetables and cereals (167). Consumption of polyphenol-rich spices such as cinnamon, cumin and ginger have anti-inflammatory effects. An intervention study providing a 3.3 g/day of a spice blend observed reduced plasma cytokine concentrations of Il-6, IL-1β, IL-8 and TNF-α when consumed for 4 weeks by healthy controls (168). Further, polyphenol compounds from green tea have also been shown to decrease pro-inflammatory cytokine levels (IL-17) and increase immunoregulatory cytokine levels (IL-10), as well as suppress the pathogenic anti-Bhsp65 antibody response in arthritis rat models (169).

In the context of inflammation, flavonoids are the most studied group of polyphenols (170). They are a wide category of polyphenolic compounds that can be found in plant-based foods including almost all fruits and vegetables. Research has focussed on flavonoids, that have several beneficial effects on the inflammatory response: inhibition of inflammatory mediators such as ROS; regulation of inflammatory enzyme activity; reduction in cytokine production and expression; and modulation of transcription factors such as NF-κB (171, 172). The therapeutic effects of flavonoids have been explored in both animal model and clinical trials for IBD. Ulcerative colitis patients who were given silymarin with standard therapy for 6 months had improved haemoglobin levels, erythrocyte sedimentation rate and disease activity compared to placebo group (173). In asthma, supplementation of pycnogenol (a proprietary mixture of water-soluble bioflavonoids) for 8 weeks showed significant improvements in serum leukotrienes compared to placebo (174). Whilst studies have looked at the benefits of flavonoids in disease, most studies have focused on animal models. Clinical trials are required to confirm the effects of flavonoids on inflammatory disease in humans.

## 4 Regulation of Inflammatory and Gastrointestinal Disease by Diet-Microbiota Interactions in the Bowel

In addition to directly interacting with immune cells and receptors, diet indirectly affects inflammation *via* modulation of the gut microbiota. Dietary fats, proteins, carbohydrates and other micronutrients all contain components that can act as substrates for microbiota (175). Non-digestible carbohydrates (i.e., dietary fibres) are a major energy source for gut microbiota, with alterations in consumption of dietary fibre observed to change the structure and function of gut microbiota within days (176). Species such as *Roseburia* and *Faecalibacterium*, more abundant in those who consume dietary fibre-rich foods (e.g. legumes, cereals, fruits and vegetables), have been associated with lower intestinal inflammatory markers, suggesting that dietary modulation may indirectly regulate inflammation *via* gut microbes (177).

**Figure 2:**
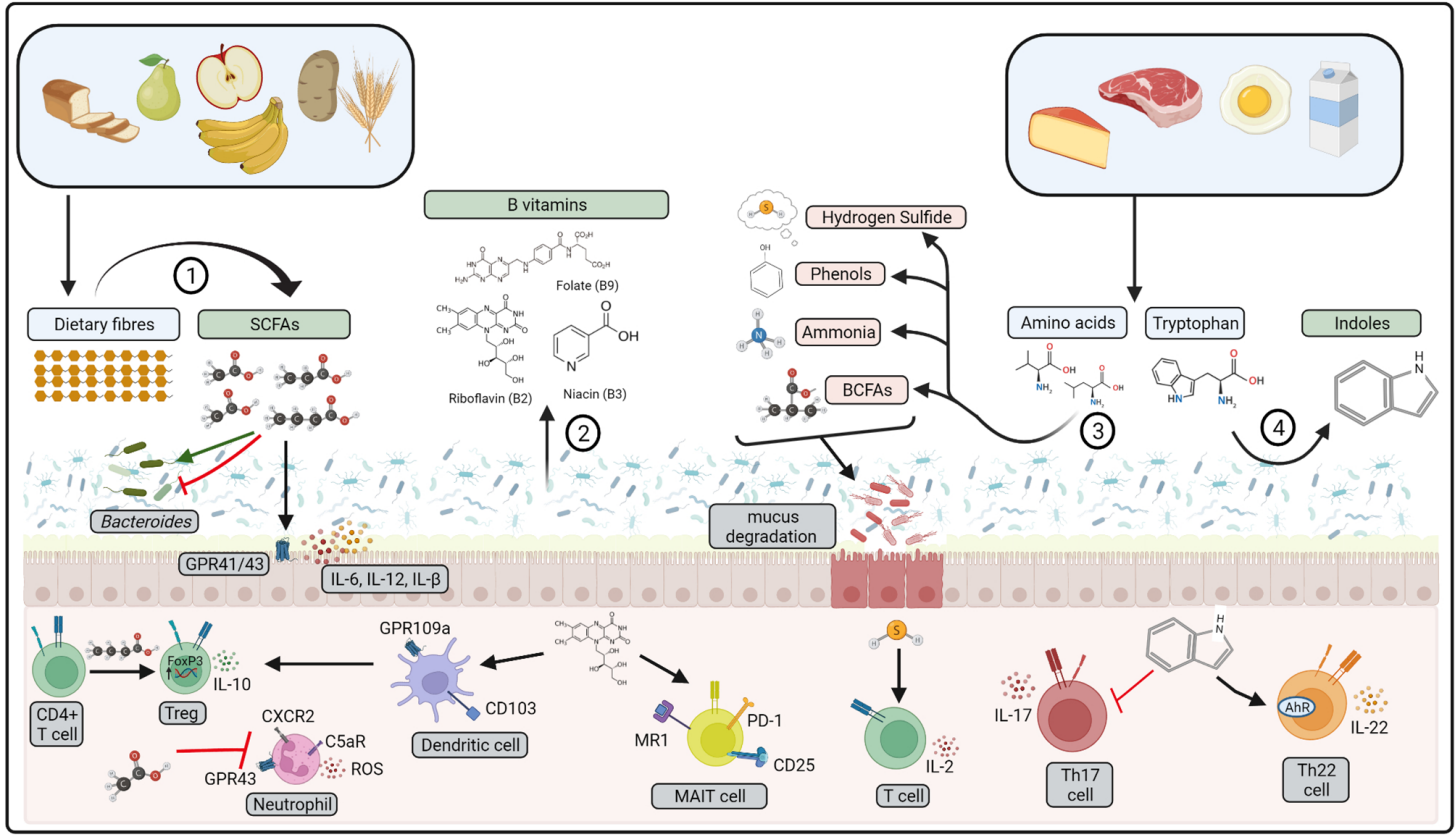
Bioactive compounds produced by gut microbiota. 1) Dietary fibres are fermented by gut microbiota to produce short-chain fatty acids (SCFAs), 2) B vitamins may be produced from gut microbiota metabolism. 3) Amino acids from dietary proteins may be fermented to produce branched-chain fatty acids (BCFA), ammonia, phenols, and hydrogen sulfide. 4) Dietary tryptophan is metabolised by gut microbiota to indoles. Pro-inflammatory compounds represented in red, anti-inflammatory represented in green. These compounds affect host physiology *via* interactions with gut microbiota, colonic epithelial cells and mucosal immune cells. ROS: reactive oxygen species, MAIT, mucosal-associated invariant T cell. Created with BioRender.com.

Dietary fibre modulates local immune homeostasis in the gut, with high-fibre diets protective in murine models of IBD (178). Furthermore, reduced incidence of Crohn’s disease is associated with increased fibre intake, particularly fibre derived from fruits (179). Anti-inflammatory effects may be directly mediated by dietary fibres that form carbohydrate structures that harbour intestinal pathogens, blocking interaction with colonic epithelial cells (180). However, the most significant interactions are with gut microbiota, that utilise dietary fibres as an energy source, promoting growth of *Bifidobacterium and lactobacillus* species (181). In the absence of dietary fibre, gut microbiota may begin to utilise mucan glycans as an alternative energy source, resulting in degradation of the mucus layer and subsequent inflammation (182). In addition, microbial fermentation of dietary fibres produces metabolites such as short-chain fatty acids (SCFA) that promote colonic homeostasis (**Figure 2**). SCFA are a key energy source that maintains colonic epithelial cell structure and function, whilst also regulating production of IL-8, IL-17, IL-1β, IL-6, IL-12 and TNF-α by colonic epithelial cells (183). In addition, SCFA have a range of immuno-regulatory properties, as discussed in more detail below. A recent study has also demonstrated that the microbial-derived SCFA butyrate may regulate the composition of *Bacteroides* in the gut, inhibiting growth of species depending on the sugar substrate available in the environment (184). This occurs in *Bacteroides* species that have increased expression of Acyl-CoA transferase and reduced expression of Acyl-CoA thioesterase, resulting in an accumulation of the toxic butyrate metabolite butyryl-CoA within the cell. Butyrate toxicity may also exacerbate chronic colitis in mice given high fructo-oligosaccharide diets, demonstrating that high-fermentable fibres may induce pro-inflammatory effects when provided at high-doses (185).

Emerging evidence has also shown that dietary-mediated modulation of gut microbiota may also regulate inflammation systemically. High fibre diets are protective in animal models of type 1 diabetes, asthma and arthritis, elicited by an increase in Treg cells (186–188). Immune changes may occur rapidly in response to alterations in gut microbiota. Native Africans who underwent a high-fat, low-fibre ‘American’ dietary intervention had increased mucosal inflammation after 2-weeks and changes to microbial metabolites (30). In contrast, high fat diets are associated with an increase in *Bilophia* and *Desulfovibrio*, as well as increased gut permeability, which drives chronic inflammation in rodents (189, 190). Indeed, high fat diets exacerbate disease in models of systemic inflammatory conditions, such as experimental autoimmune encephalomyelitis (191). Similarly, mice fed a high sugar diet have increased colonic IL-1β and TNF-α and develop more severe colitis in response to DSS treatment (192). Sugar-microbiota interactions exacerbate inflammation in mice fed 10% glucose, increasing the abundance of *Akkermansia muciniphilia* and *Bacteroides fragilis* that degrade the colonic mucus lining (193). Fructose-induced mucosal inflammation may result from dysregulated intracellular protein transport and secretion, due to a build-up of toxic fructose-metabolites within colonic epithelial cells (194). Indeed, this also dysregulates the production of tight junction proteins claudins and occludin, causing endotoxemia and liver inflammation. Furthermore, mice fed high fat diets have increased neutrophils, macrophages, and pro-inflammatory cytokines in the lung after sensitisation and exposure to house-dust mite, highlighting that a gut-lung immune axis may be partially mediated by diet (195). The pro-inflammatory environment induced by a high fat diet may promote the differentiation Ly6C^+^ monocyte precursors into inflammatory macrophages, that migrate to the lung and worsen pathology (196). Conversely, a high fibre diet was shown to reduce airway inflammation and the number of inflammatory neutrophils and cytokines in the lungs (197).

Diet-induced changes to gut-physiology will also affect the structure and function of the gut microbiota, which in turn affect inflammation. Many of these changes occur locally along the gastrointestinal tract in response to meals. Consumption of a high-fat meal will result in increased bile acid secretion, which has antimicrobial properties, particularly on gram-positive phyla such as Bacteroidetes and Actinobacteria (198). Bile acid secretion and its subsequent metabolism to sulfur-rich taurocholic acids promote growth of *Bilophila wadsworthia*, a gram-negative species that has been associated with gut inflammation (199, 200). There may also be an association between gut-physiology and the complexity of microbes within the bowel. For example, a positive correlation between gut transit time and microbiome alpha diversity was observed in healthy individuals, with increased abundance of *Akkermansia muciniphilia* in those considered to have longer transit time (> 59 hours) (201). Although acute consumption of non-fermentable fibres may hasten transit time, it remains unclear if this may result in long-term changes to the gut microbiota and if this alters immune homeostasis (202).

Additional components of processed foods, such as emulsifiers or additives may also directly affect gut physiology, by integrating with the mucus lining of the gut (203). Consequently, this may promote encroachment of bacteria to the gut epithelial lining by physically altering the mucosal layers that normally protect the gastrointestinal epithelial cells. The addition of a high-dose (50 mg/kg(body weight)/day) of food additive titanium dioxide to the drinking water of mice resulted in colonic inflammation driven by CD8^+^ T cells and macrophages (204). Although, it has not yet been established if a similar phenomenon occurs in humans who ingest high levels of additives or emulsifiers, consumption of a high dose (15 g/day) of the synthetic emulsifier carboxymethylcelluose (CMC) for 11 days has been observed to alter faecal metabolome and increase microbial encroachment on the epithelium in healthy subjects (205) In contrast, dietary minerals such as calcium phosphate have buffering properties within the gut, precipitating toxic bile acid metabolites and as a consequence maintaining microbial homeostasis (206). Taken together, food additives and minerals are likely to play a role in regulating gut microbiota in addition to major macronutrient substrates. This is particularly important in IBD, where breakdown of the mucosa and mucus layer exacerbates disease activity and chronic inflammation.

### 4.1 Gut Microbiota Metabolites With Immunoregulatory Properties

#### 4.1.1 Short Chain Fatty Acids

SCFAs are 2-4 carbon chain organic acids produced from the fermentation of dietary fibre by gut microbiota. These are predominantly acetate, propionate and butyrate found most abundantly in the colon, at 10-100 mmol/L (207). SCFAs maintain colonic epithelial homeostasis, act as a major energy source for colonocytes and directly regulate inflammatory cytokine production by colonic epithelial cells (208, 209). Additionally, they can regulate activity of macrophages, neutrophils, dendritic cells, T cells and B cells within the colonic mucosa (183). SCFAs may modulate immune cell activity *via* engagement of SCFA specific G-protein coupled receptors on the cell surface, or within the cell by modulating epigenetic regulation by inhibiting histone deacetylases (HDAC) (197, 210). Butyrate may inhibit HDAC in human cell lines at 10-100 µmol/L, with propionate and acetate showing similar properties at 100-1000 µmol/L (211, 212). SCFA-mediated HDAC inhibition may result in altered expression of transcriptional regulators of immune function, depending on the SCFA concentration present and surrounding immune milieu. Butyrate-mediated inhibition of HDAC9 and HDAC6 in T cells drives expression of FOXP3 and differentiation to a Treg phenotype when provided to mice at 100-200 mM in steady-state conditions (213). In contrast, HDAC inhibition by butyrate drives T cells to express Tbet and differentiate to a Th1 phenotype in the presence of IL-12 (212).

SCFAs are also processed intracellularly to form metabolic intermediates such as acetyl-CoA that drive metabolic and epigenetic changes upon immune cell activation (214). Immune effects of SCFAs may also be found systemically as they can be absorbed into the peripheral circulation *via* portal veins within the gut and can be detected at a serum concentration of 2-500 µmol/L (207). Indeed, potent systemic anti-inflammatory effects have been observed when SCFAs are given to mice in models of inflammatory disease and allergy (186, 197, 215, 216). This has been purported to act *via* downregulation of pathogenic responses driven by B cells, Th1, Th2 and Th17 subsets with concurrent upregulation of Treg responses. In addition, butyrate has recently been reported to promote regulatory B cells and inhibit germinal centre and plasmablast formation, reducing severity of antigen-induced arthritis in mice (217).

#### 4.1.2 Protein Catabolites

Breakdown of dietary protein by gut microbiota produces protein catabolites such as branched-chain fatty acids (BCFA), ammonia, phenols and hydrogen sulfide (**Figure 2**) (218). Protein catabolites may increase in response to increased dietary protein consumption, particularly if derived from animal protein sources (219). Amino acid breakdown are driven by species of *Escherichia*, *Eubacterium*, *Clostridium* and *Enterococcus (* 220, 221), of which *Clostridium perfringens* and *Escherichia coli* may become opportunistic pathogens in a state of dysbiosis (222, 223). Indeed, mice fed a high protein diet have reduced colonic IgG and increased prevalence of *E.coli* in the gut (224). Colonic epithelial cells exposed to ammonia and hydrogen sulfide have impaired growth leading to increased colonic epithelial cell turnover, upregulated pro-inflammatory cytokine production and consequent increased epithelial permeability (185, 225, 226). Furthermore, hydrogen sulfide enhances T lymphocyte activation and IL-2 production upon T cell receptor (TCR) stimulation, through interaction with the actin and tubulin cytoskeleton (227). Hydrogen sulfide treated mice had increased L-1β, IL-6, TNF-α, MCP-1, and MIP-2 in plasma, lung and liver after caecal ligation and puncture (CLP)-induced sepsis (228). In contrast, hydrogen sulfide treatment enhanced resolution of colonic inflammation in mice, indicative of an anti-inflammatory role within the colon (229). This may be *via* promotion of FOXP3^+^ Tregs, driven by enhanced expression of methylcytosine dioxygenases Tet1 and Tet2 by hydrogen sulfide (230). Secondary metabolism of BCFA by microbes such as *Bacteroides fragilis* can also produce lipid α-galactosylceramides. that regulate the function and number CD1d restricted natural killer T cells in murine gut tissue (231).

Immune regulation may also be driven by tryptophan metabolites produced *via* microbial metabolism of tryptophan. These include indole and its derivatives; indole-3-aldehyde (IAld), indole-3-acid-acetic (IAA), indole- 3-propionic acid (IPA), indole-3-acetaldehyde (IAAld), and indoleacrylic acid (232). These compounds form ligands to activate the aryl-hydrocarbon receptor (AhR) found within gut epithelial cells, intraepithelial lymphocytes, Th17 cells, macrophages, neutrophils and dendric cells (233). Engagement of AhR within intraepithelial CD4^+^ T cells, γδ T cells and innate lymphoid cells increases production of IL-22 whilst downregulating IL-17 in mice (234, 235). Taken together, this results in mucosal immune tolerance to gut microbiota and maintenance of gut epithelial integrity, thus preserving intestinal homeostasis. Indeed, gut microbiota from patients with Crohn’s disease and coeliac disease may have a reduced capacity to metabolise tryptophan, leading to defective AhR activation (236, 237). Tryptophan metabolite activation of AhR may also promotion of B-regulatory cells that ameliorate systemic inflammation in a mouse model of arthritis (217).

Microbial degradation of immunogenic proteins, such as gluten and amylase trypsin inhibitors, may also directly affect the T cell and innate immune response in coeliac disease. Gluten-peptides produced by *Pseudomonas aeruginosa* have increased translocation across the mucosal barrier in mice, whilst also inducing stronger responses from T cells derived from patients with coeliac disease (238). In contrast, microbial degradation of gluten and amylase trypsin inhibitors by *Lactobacillus* species produces peptides that are less immunogenic to coeliac disease patients and gluten-sensitised mice, highlighting that these species may be protective against inflammation in the context of coeliac disease (238, 239).

#### 4.1.3 Vitamins

A range of *Lactobacillus, Bacteroides* and *Bifidobacteria* species can synthesise water-soluble B vitamins such as riboflavin (Vitamin B2), niacin (vitamin B3) and folate (vitamin B9) (240). Unlike dietary derived vitamins that are mainly absorbed by the host along the small intestine, microbial-derived vitamins are absorbed in the colon where they may interact with mucosal immune cells (**Figure 2**) (241). Vitamin B2 metabolites are crucial for regulating host defence, as mucosal-associated invariant T (MAIT) cells recognise Vitamin B derivatives presented *via* the MHC like protein (MR1) on antigen presenting cells in the colon (242). MAIT cells may directly respond to changes in bacterial growth by responding to changes in the abundance of riboflavin, increasing expression of CD69, CD25 and PD-1 expression in response to stimulation with *E. coli* in growth phase (243). In mice, niacin may also signal to colonic CD103^+^ dendritic cells through engagement of the cell surface receptor GPR109a, to promote a T-regulatory phenotype upon engagement of T cells (244). Taken together, this highlights a significant role for microbial compounds in regulating immune homeostasis within the colon.

## 5 Utilising Dietary Therapy in the Treatment of Human Gastrointestinal and Inflammatory Disease

To date, the clinical use of dietary therapy to treat human inflammatory disease has shown the most efficacy in patients with coeliac disease, in which pathogenesis is inherently linked to consumption of wheat. Following a strict gluten-free diet improves coeliac disease pathology characterised by a reduction in intestinal damage, steatorrhea, diarrhoea and weight loss (245, 246). However, there is an emerging body of evidence that highlights clinical value in other gastrointestinal and peripheral inflammatory diseases.

### 5.1 Gastrointestinal Disease

Dietary therapy in a form of exclusive enteral nutrition (EEN) has long been considered as the first-line approach to induce remission in active paediatric Crohn’s disease preferable to corticosteroid treatment (247, 248). This liquid-based product is formulated to deliver all essential macronutrient and micronutrients, without the need for consumption of any whole-foods. EEN is thought to induce anti-inflammatory effects in the gastrointestinal tract by eliminating most food-derived antigens and substrates, consequently reducing the activity of both the immune system and the resident-microbiota (249). Although some EEN formulas may also contain emulsifiers such as carboxymethyl cellulose (CMC) and polysorbate 80, implicated above to trigger inflammation in pre-clinical models, no differences in disease remission rates have been observed between those that containing these food additives and those that do not (250). Hence, the amount of food additive delivered over the duration of a EEN course is unlikely to trigger disease. Adult Crohn’s disease patients often have poor compliance to EEN over a long period due to poor palatability with good compliance only achieved through delivery *via* a nasogastric tube (251). Nevertheless, more evidence is accumulating to suggest EEN is also effective in treating mucosal inflammation in adult Crohn’s disease patients (252, 253).

In addition, whole-food dietary interventions have been explored as alternative treatment options as summarised in **Table 2**. Some of these therapeutic diets involve exclusion of many food groups:

**Table 2:** Dietary randomized control trials to treat Inflammatory bowel disease.

Diet	Study design	Patient cohort	No.	Duration	Primary endpoint	Secondary endpoints	Ref.
Crohn’s disease exclusion diet	Observational	CD patients unresponsive to biologics	21 (11 Adult, 10 children)	12 weeks	Clinical response (remission ≤3 HBI) 19/21 (90.4%)	Clinical remission 13/21 (62%) ↓ HBI *(*P < 0.001) ↓ CRP (P=0.02)	(254)
	Observational	Paediatric + young adult CD patients with active disease	47	6 weeks	Remission (≤3 HBI): 70.2%	↓ PCDAI (P < 0.001), ↓ HBI (P < 0.001) ↓ CRP (P < 0.001)	(255)
	RCT	Paediatric CD patients	74	12 weeks	↑ Tolerance: CDED+PEN vs. EEN (P* < 0.01)*	↑ Corticosteroid-free remission: CDED+PEN vs. EEN (P=0.01) ↔ Faecal calprotectin CDED+PEN vs. EEN (P=0.43)	(256)
	RCT	Adult CD patients with mild/moderate disease	40	24 weeks	↔ Remission ( < 5 HBI) at wk 6: CDED+PEN vs. CDED (P=0.46)	↔ Clinical remission at Wk24 (P=0.11) ↔ endoscopic remission at Wk24 (P=0.74) ↔ CRP (P=0.79), Faecal calprotectin (P=0.60)	(257)
Specific carbohydrate diet	Observational	Paediatric CD and UC patients with active disease	12 (10 analysed)	12 weeks	Remission (PCDAI/PUCAI < 10): 80%	↓ PCDAI (-23.5)^a^ ↓ PUDAI (-21.6)^a^ ↓ CRP^a^, Faecal calprotectin^a^	(258)
Low FODMAP diet	RCT	Quiescent CD and UC patients	52 (43 analysed)	4 weeks	↔ IBS symptom score: Low FODMAP: -67 vs. Control: -34 (*P=*0.075)	↔ disease activity (P=0.8) ↑ IBD control score (P=0.03) ↔ CRP (P=0.25), Faecal calprotectin (P=0.98), peripheral blood T cells	(259)
	RCT	Quiescent/mild CD and UC patients	55	6 weeks	↔ disease activity (HBI/mayo score): Low FODMAP vs. control diet (CD patients, P=0.28, UC patients, P= 0.84):	↔ faecal calprotectin (P*=*0.13) ↔ CRP (P=0.64) ↔ IBD control score (P=0.89)	(260)
Mediterranean diet	Observational	CD patients	58	6 months	↓ BMI: -0.48, P=0.032	↑ QoL (P < 0.001) ↓ CRP (P=0.04) ↓ Faecal Calprotectin (P=0.04)	(261)
	Observational	UC patients	84	6 months	↓ BMI: -0.42, P=0.002	↑ QoL (P < 0.001) ↓ CRP (P=0.01) ↓ Faecal Calprotectin (P=0.049)	(261)
	RCT	CD patients with mild/moderate disease	191	12 weeks	↔ Symptomatic remission (sCDAI < 150 at week 6): Mediterranean diet vs. SCD (P=0.77)	↔ clinical remission (P=0.28) ↔ CRP (P=ns) ↔ Faecal Calprotectin (P=0.44) ↔ QoL (P>0.3)	(262)
Low Fat, High fibre	RCT	Quiescent UC patients	18 (17 analysed)	4 weeks	↑ QoL: Low fat/high fibre vs. control diet (P=0.04)	↔ CRP, Faecal Calprotectin (P=ns) ↓ serum amyloid A (P=0.07)	(263)

RCT, randomised control trial; HBI, Harvey-Bradshaw index; CRP, C-reactive protein; CDED, Crohn’s disease exclusion diet; PEN, partial enteral nutrition; QoL, Quality of life; PCDAI, Paediatric Crohn’s disease activity index; PUCAI, Paediatric ulcerative colitis activity index. UC, ulcerative colitis.

a no p-value reported.

↑, increased; ↓, decreased; ↔, unchanged; NS, not significant.

- Crohn’s disease exclusion diet: heavily restricted diet, designed to be consumed alongside partial enteral nutrition. Patients exclude intake of foods such as dairy, wheat, emulsifiers, maltodextrins and processed foods that may stimulate the gut microbiota and mucosal immune system (255). It is also low in animal fat with limited intake of fruits and vegetables.
- Specific carbohydrate diet: excludes all grains, sugars (except honey), processed foods, and dairy, aside from specific fermented yogurt and some hard cheeses (258).
- Low FODMAP (Fermentable Oligosaccharides, Disaccharides, Monosaccharides And Polyols) diet: excludes fermentable carbohydrates (fructans, oligosaccharides, disaccharides, monosaccharides and polyols), that if malabsorbed in the small intestine, undergo colonic fermentation by the microbiota and trigger luminal distension (264). It is traditionally used to treat patients with irritable bowel syndrome to relieve gastrointestinal symptoms (e.g., wind/bloating) and has recently been explored for its potential benefits in management of Crohn’s disease.

Although there have been some clinical improvements reported in prospective observational studies utilising these exclusion diets to treat IBD patients, many of these studies are difficult to compare with diverse clinical endpoints, primarily using subjective symptom-based scoring systems, patient populations and durations. Results of many other randomised control trials may also fail to reach statistical significance when compared to control diets, that can induce clinical response compared to patient habitual diets (265). Levine et al. reported that the Crohn’s disease exclusion diet resulted in significantly greater corticosteroid-free remission amongst paediatric Crohn’s disease patients when compared to exclusive enteral nutrition (256). However, another recent randomised trial found no difference in patient clinical remission rates between the Crohn’s disease exclusion diet when compared to partial enteral nutrition with Crohn’s disease exclusion diet, highlighting the importance of choosing an appropriate comparator (257).

Indeed, there may also be clinical value in utilising a traditional dietary pattern as opposed to a restrictive diet. A recent randomised clinical trial by Lewis et al. showed similar clinical or inflammatory responses in patients who followed a Mediterranean diet and specific carbohydrate exclusion diet (262). Besides EEN, the current evidence suggests that these dietary interventions being explored as potential treatments for IBD seem to relieve gastrointestinal symptoms. However, whether they have roles in ameliorating mucosal inflammation requires further evaluation. This is particularly the case for the low FODMAP diet, which has been found to reduce gastrointestinal symptom scores without improving clinical disease scores or biomarkers in quiescent IBD patients (259). To date, there is limited data on the effect of altering FODMAPs on inflammation in the gastrointestinal tract. One preclinical study found that colonic cytokine levels and histology scores were similar in mice provided with low and high FODMAP diets after induction of acute colitis (266). In contrast, supplementation of FODMAPs, particularly fructo-oligosaccharides (FOS) has shown limited success in reducing disease scores in patients with active Crohn’s disease (267). However, supplementation of 15 g/day FOS may induce gastrointestinal symptoms in patients resulting in poor tolerance (268). Indeed, further studies are required to identify which subsets of patients may most benefit from fibre-supplementation.

Beyond IBD, exclusive elemental diet is well established as a treatment for eosinophilic oesophagitis (269). The disease pathophysiology of eosinophilic oesophagitis is poorly understood, however it is thought to be caused by a breakdown in oral tolerance (270). Food antigens may trigger Th2-mediated inflammation, releasing IL-5, IL-13 and IgE. This promotes the recruitment and degranulation of eosinophils and mast cells thought to contribute to tissue damage. Patients who follow an exclusive elemental diet have reduced symptoms, endoscopic and microscopic signs of inflammation (271). An elimination diet approach may also be used to treat eosinophilic oesophagitis. However, this approach may not be as effective as an elemental diet due to the wide range of food allergens that trigger the allergic response (272, 273).

### 5.2 Peripheral Inflammatory Diseases

There has been limited success in using dietary modification to treat inflammatory skin conditions. Exclusive enteral nutrition followed by food reintroduction was found to improve refractory atopic eczema in a small cohort of children, although 27% of the cohort did not respond to the treatment (274). Indeed, a systematic literature review of randomized controls trials to assess the efficacy of dietary exclusion found it had no significant benefit in the treatment of atopic eczema (275). Vitamin D supplementation may reduce skin inflammation in subsets of atopic dermatitis patients who have recurrent bacterial infection and pre-existing vitamin D deficiency, however further placebo-controlled randomised control trials are needed to confirm these effects in other cohorts (276). A randomised clinical trial found that patients with asthma and vitamin D deficiency had significantly improved asthma control after 6 months of weekly oral calcifediol (1,25 dihydroxy vitamin D3) supplementation compared to placebo (277). Indeed, long-term supplementation with vitamin D may also be protective against inflammatory disease. A recently completed randomized controlled trial showed that supplementation of vitamin D, with or without omega-3 fatty acids, for five years significantly reduced the incidence of developing autoimmune diseases (e.g. rheumatoid arthritis, systemic lupus and psoriasis) in a large cohort of older adults, compared to no supplementation (278).

Several small dietary intervention trials have also been conducted in patients with other inflammatory diseases. A study using a small group of multiple sclerosis patients (n=20) found that those who consumed a Mediterranean-style diet, high in fruits and vegetables, had reduced IL-17 expressing CD4^+^ T cells and lower clinical symptom scores after 12 months (279). A significant increase in the abundance of *Lachnospiraceae* was also found, that correlated with further changes to CD14^+^ monocytes and FOXP3^+^ Tregs, suggesting a role for the gut microbiota in driving immune change. Indeed, significantly lower plasma SCFA concentrations were seen in another cohort of multiple sclerosis patients when compared to healthy controls (280). Furthermore, a cohort of 29 rheumatoid arthritis (RA) patients who consumed a high-fibre bar each day for 30 days had significantly reduced serum MCP-1, IL-18 and IL-33, potentially due to an increased delivery of systemic SCFA (281). However, no changes to clinical indices were reported. Another high-fibre intervention study in 31 RA patients found increased Th1/Th17 ratio and Treg cells after 28 days of consumption (282). Decreased markers of bone erosion were also reported, suggesting that high-fibre intervention may have clinical relevance in RA.

Resistant starch that has been chemically modified to release additional acetate and butyrate in the colon has recently been reported to induce a regulatory immune phenotype in a small cohort of Type-1 diabetes patients (283). Consumption of 40 g/day of chemically modified high-amylose maize starch for 6 weeks increased the proportion of peripheral blood Treg cells, CTLA-4 expressing T cells and reduced the proportion of CD86 expressing on dendritic cells. Although, this did not significantly improve glucose control or insulin requirements. Similarly, consumption of 12 g of fibre supplement inulin for 7 days was found to reduce sputum eosinophils and improve asthma symptom control in a group of 17 asthmatics (284). Studies in larger patient cohorts are required to clinically validate favourable anti-inflammatory effects observed in these pilot studies.

### 5.3 Fermented Foods in the Treatment of Inflammatory Disease

**Figure 3:**
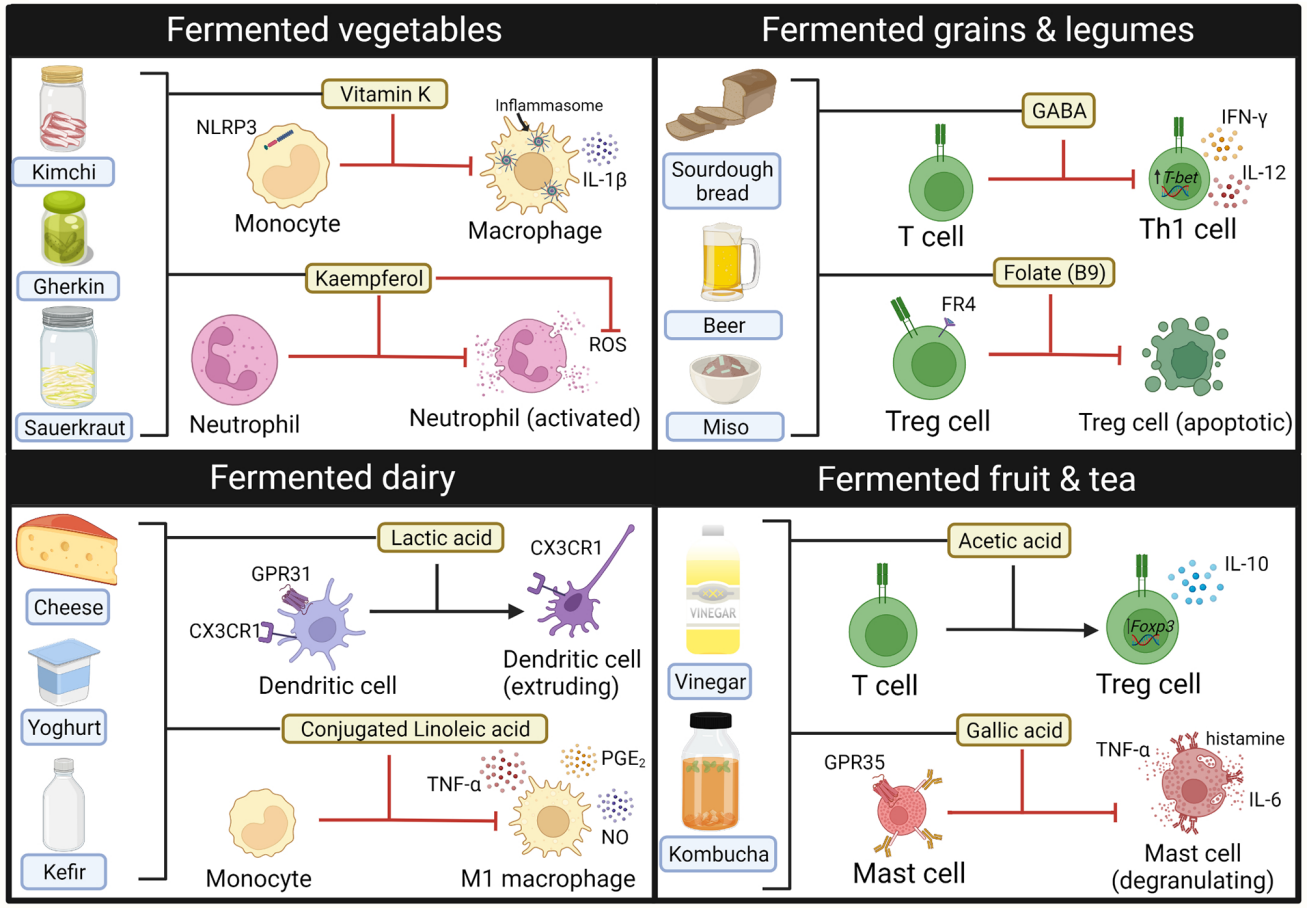
Immune modulating compounds contained within Fermented foods. Bioactive compounds from fermented foods that have anti-inflammatory effects on the activity and phenotype of innate (macrophages, neutrophils, mast cells) and adaptive (T-cells) immune cells. GABA, gamma-aminobutyric acid. Created with BioRender.com.

Using fermented foods to deliver probiotics and beneficial microbial metabolites (i.e., postbiotics) has been of recent interest, particularly as many fermented food products are readily available to the public. Despite these products being marketed as anti-inflammatory, there is currently limited clinical evidence to support this. Consumption of fermented dairy products such as yoghurt has been associated with reduced serum CRP in healthy people, although this association may have been confounded with overall diet quality (285). Indeed, fermented foods contain a wide range of compounds with immunomodulatory properties when exposed to cells *in vitro* (**Figure 3**) (213, 286–292). Consumption of fermented foods could deliver immune-modulatory compounds such as SCFA systemically, particularly if consumed multiple times per day (293). They also contain a diverse range of probiotic bacteria that initiate fermentation, such as lactic acid bacteria, acetic acid bacteria and *Bacillus* species [reviewed elsewhere (294)]. Healthy participants who followed a high fermentable food diet for 10 weeks were found to have reduced serum levels of inflammatory markers such as Il-6, IL-18 and CXCL10, highlighting potential immunomodulatory effects of such foods (295). However, it is also important to consider the fungal component of fermented foods, that may have the potential to become opportunistic pathogens in the context of dysbiosis. *Debaryomyces hansenii* is a fungus contained in many fermented cheeses that may be enriched in inflamed intestinal tissues from Crohn’s disease patients. Isolates of *D.hansenii* inhibit intestinal crypt regeneration in mice models of intestinal injury, suggesting that they may be pathogenic (296). Taken together, further studies are needed to elucidate if consumption of fermented foods could have clinical benefit in the disease setting.

### 5.4 Challenges of Translating Dietary Therapy to Clinical Practise

Indeed, there is a clear need to design appropriate clinical trials to examine if the powerful anti-inflammatory effects of dietary modifications in pre-clinical models of disease can be translated in the clinical setting. Dietary modifications in animal models are often extreme, with large doses of fats or fibre utilised. The amount of fibre in a high-fibre diet given to a mouse has been estimated to be equivalent to 274 g/day for a human, nearly 10 times that of the recommended daily intake (297). Furthermore, comparator diets often do not contain any of the macronutrients of interest (e.g., no-fibre or no-fat diets) which are not realistic for human consumption. Although some human intervention studies have highlighted that extreme dietary changes, such as all-meat or all-vegetable diets may alter the composition of the gut microbiota within a matter a days, these are unlikely to be suitable for long-term consumption as they would lead to deficiencies in vital micronutrients (176). Indeed, short-term dietary intervention is also unlikely to induce any significant changes to inflammation. Consumption of a 5-day high fibre diet (39 g/day) failed to change plasma cytokine levels or circulating Treg cell frequency despite increasing plasma SCFA levels in healthy volunteers (298). Therefore, longer-term interventional studies over weeks to months are required to observe significant changes. Expression levels of IL-1β, IL-18, TLR-2 and TLR-4 were significantly decreased in individuals with Type 2 diabetes who consumed oral SCFA for 45 days, suggesting that this may be a more optimal timeframe for immune modulation (299). However, the gold-standard for dietary studies is for a complete intervention which may be difficult for participants to adhere to for an extended period (300). A careful trade-off must be made to design studies of sufficient duration and dose to induce change with an adequate level of control within a realistic timeframe.

The correct patient subset must also be identified for dietary intervention. This is particularly important in IBD, as patients with active disease may be less likely to adhere to a strict dietary intervention if experiencing symptoms (301). Elimination diets may only be suitable to treat inflammatory disease in those who also have an underlying allergy. For example, the use of a gluten-free diet has been weakly recommended for psoriasis patients who also test positive for serologic markers of gluten sensitivity (302). It may also be difficult to control for other lifestyle and socioeconomic factors, such as exercise and access to fresh foods attributed to anti-inflammatory effects. Furthermore, low socioeconomic status may be correlated to poor diet quality and has also been recently linked to exacerbated COVID-19 infection (14). There is a need for public health initiatives to improve education and accessibility to fresh produce, so that those who may be most in need of nutritional improvement can do so. This may be particularly critical during pregnancy, as maternal intake of vegetables and grains have been found to be preventative against development of asthma and allergic rhinitis in young children (303). This may be *via* epigenetic modification of the thymic microenvironment to promote Treg cells that protect against Th2 cytokine responses, as has been observed in murine models of allergic airways disease (197, 304).

## 6 Conclusion

There is now a large body of evidence implicating diet in the development of inflammatory and gastrointestinal disease. This has been built upon epidemiological associations that identify a high-fat and low fibre western diet, as a risk factor for the development of inflammatory disease, particularly when compared to traditional diets. These effects may be driven by direct nutrient-immune system interactions with fats, proteins, vitamins, minerals and polyphenols. Furthermore, nutrients also modulate the structure and function of the gut microbiota, that indirectly affects local and systemic inflammation. Although dietary modulation has powerful effects on inflammation in animal models of human inflammatory disease, there have been challenges translating these observations into humans. This is due to inherent differences in physiology between rodents and humans, as well as extreme diets utilised in animal models that often use zero-nutrient diets as comparators.

Despite specific dietary therapies that have shown success in treating coeliac disease and IBD, there remains a paucity of data to show if dietary therapy may have clinical relevance in other human inflammatory disease. Recent data from pilot studies utilising dietary fibre supplements and fermented foods have shown encouraging anti-inflammatory and clinical effects in patients with other inflammatory conditions. However, it remains to be seen if findings from these pilot studies may be replicated in larger patient cohorts. Future studies must adequately control for background diet, whilst also providing a dietary intervention that is tolerable for patients over a long period. Further advances in microbiome and immunological profiling may allow for more accurate identification of patient populations that will most benefit from dietary interventions. Ultimately, collaboration between immunologists, clinicians, nutritionists, and dietitians is required to design appropriate clinical trials to confirm clinical efficacy of dietary therapy to treat inflammatory disease.

## Author Contributions

PG and SI reviewed the literature and drafted the manuscript. AS, TK, and FR critically reviewed and edited the manuscript. All authors read and approved the final manuscript.

## Funding

TK is funded by a UK Medical Research Council (MRC grant MR/R001901/1). PG is funded by a research grant by Imhotex LTD. The funder was not involved in the study design, collection, analysis, interpretation of data, the writing of this article or the decision to submit it for publication.

## Conflict of Interest

The authors declare that the research was conducted in the absence of any commercial or financial relationships that could be construed as a potential conflict of interest.

## Publisher’s Note

All claims expressed in this article are solely those of the authors and do not necessarily represent those of their affiliated organizations, or those of the publisher, the editors and the reviewers. Any product that may be evaluated in this article, or claim that may be made by its manufacturer, is not guaranteed or endorsed by the publisher.
